# Heart Rate Variability Biofeedback Efficacy on Fatigue and Energy Levels in Fibromyalgia: A Secondary Analysis of RCT NCT0412183

**DOI:** 10.3390/jcm13144008

**Published:** 2024-07-09

**Authors:** Mauro Giovanni Carta, Giulia Cossu, Diego Primavera, Cesar Ivan Aviles Gonzalez, Giorgia Testa, Serena Stocchino, Gabriele Finco, Maria Teresa Littera, Maria Cristina Deidda, Stefano Lorrai, Clelia Madeddu, Antonio Egidio Nardi, Federica Sancassiani

**Affiliations:** 1 Department of Medical Sciences and Public Health, University of Cagliari, 09042 Cagliari, Italy; maurogcarta@gmail.com (M.G.C.); serena-stocchino@hotmail.it (S.S.); stefanolorrai@live.it (S.L.); federicasancassiani@yahoo.it (F.S.); 2 Department of Pedagogy, Psychological Sciences and Philosophy, University of Cagliari, 09042 Cagliari, Italy; 3 Center for Palliative Care and Pain Management, University Hospital of Cagliari, 09042 Cagliari, Italy; mc.deidda@outlook.com; 4 Institute of Psychiatry-IPUB, Federal University of Rio de Janeiro, Rio de Janeiro 20010-90, Brazil; antonioenardi@gmail.com

**Keywords:** fibromyalgia, biofeedback, heart rate variability, fatigue, perceived energy, advanced technology laboratory, RCT

## Abstract

**Background**: Fibromyalgia syndrome (FMs) is a chronic condition characterized by widespread musculoskeletal pain and a range of complex symptoms, with chronic fatigue being a central feature significantly impacting daily life. The aim of this study was to analyze the secondary outcomes, specifically those related to perceived energy and fatigue symptoms in a randomized controlled trial (RCT) assessing the efficacy of heart rate variability biofeedback (HRV-BF) as an adjunctive treatment for FMs. **Methods**: Sixty-four FMs patients were randomly assigned to either receive 10 HRV-BF training sessions alongside standard pharmacological therapy (experimental group) or standard therapy alone for 10 weeks (control group). For this secondary analysis, potential improvements in specific items were evaluated regarding perceived energy (Item 10 of the Short-Form Health Survey), the ability to walk and climb stairs (Item 7 and Item 11 of the Fibromyalgia Impact Questionnaire, respectively), and the impact of pain on movement ability (Item 17 of the Bodily and Emotional Perception of Pain). **Results**: The experimental group demonstrated an improvement in the perception of energy, the ability to walk, and the impact of pain on movement ability. However, the same improvement was not observed in the ability to climb stairs. **Conclusions**: Fatigue assessment has emerged as a crucial factor for evaluating treatment efficacy in FMs and related conditions linked to altered energy levels, such as bipolar depression, and can offer valuable insights for precisely guiding HRV-BF treatments. ClinicalTrials.gov with code: NCT04121832.

## 1. Introduction

Fibromyalgia syndrome (FMs) is a chronic condition [1,2] with a consistent prevalence that varies in community surveys, ranging from 1.6% in a French study [3] to nearly 7% in a US survey [4], with females at higher risk [5]. The clinical presentation is characterized by chronic widespread pain accompanied by symptoms such as fatigue, sleep disturbances, headaches, muscle stiffness, attention and concentration deficits [1,2], and mood disorders with a recognized profile closely resembling the characteristics of the subthreshold bipolar spectrum [6,7,8].

In the neo-Kraepelinian framework, the term encompasses a unified spectrum comprising bipolar disorders, major depressive disorders, subthreshold presentations often characterized by initial subthreshold hypomania followed by chronic depression with a component of fatigue, and non-pathological presentations featuring phases of “hyperergia/hyperactivity” followed by downturns and dysregulation of social and behavioral rhythms [9,10,11,12,13,14,15]. The shared manifestation of fatigue syndrome, a central symptom in fibromyalgia and in the bipolar spectrum, has prompted speculation regarding a potential link between these disorders [16,17,18], positing a common etiopathogenesis rooted in inflammation [17,19]. Indeed, the occurrence of stressful life events and mood symptoms concurrently exacerbates pain perception in fibromyalgia, potentially influencing treatment response [3,20].

The etiology and pathophysiology of FMs remain to be fully established. Dysfunction within the central pain processing system has been considered [5] as a potential consequence of chronic infections and inflammation [16,21]. However, circuits within the central nervous system involving glutamate and substance P, serotonin, and noradrenaline appear to be implicated [22,23]. This may be linked to the documented efficacy of antidepressants [24]. Nonetheless, in alignment with the hypothesis of a shared spectrum with bipolar disorders [25], concerns have been raised regarding the long-term efficacy of antidepressants [26] as cases of antidepressant-induced mania have been reported [27].

The current treatment options for FMs remain unsatisfactory [24], leading to high levels of healthcare utilization and increased costs [1,5]. Indeed, fibromyalgia has been identified as the most common reason for long-term job impairment, absenteeism, and sick leave from work [28]. Therefore, there is an urgent need for effective treatments.

A recent Phase II randomized controlled trial explored the potential efficacy of heart rate variability biofeedback (HR-BF) training as an adjunctive therapy for fibromyalgia [29]. The results did not conclusively demonstrate the treatment’s effectiveness. However, given the exploratory nature of the trial, it is highly likely that the insufficient power of the study resulted in a beta error. As outlined in the trial protocol, these findings will inform the calculation of a sample size to prevent such errors in Phase III trials. Additionally, the exploratory nature of the study prompted a broad exploration of potential efficacy outcomes, which indicated a large number as the primary results. Discussions with the women participating in the study led to the hypothesis that a notable improvement may have been observed in the perception of energy and, consequently, in the symptom of chronic fatigue. This symptom is primarily assessed by Item 10 of the Short-Form Health Survey (SF-12) scale [30], with the anticipated improvement being a secondary outcome of the RCT. However, it is worth noting that this symptom is also linked to other specific items on the scales used to measure the primary outcomes. Lack of energy and chronic fatigue is a significant symptom of FMs, primarily because it is considered one of the most disabling components [1].

Considering how much these elements can impact the daily lives of people suffering from FMs, we opted to conduct a secondary analysis of the trial results, with a specific focus on the hypothesis of improvement in the low energy/fatigue syndrome through HR-BF training. We selected Item 10 of the SF-12 as the main measure; however, to ensure robust verification, we also identified other potential measures of this specific outcome, taking into account the variety of instruments used in the trial.

## 2. Methods

### 2.1. Study Design

This study constitutes a secondary analysis of a 10-week randomized controlled trial (RCT) focusing on feasibility (ClinicalTrials.gov Identifier: NCT04121832). While the primary analysis of the study highlighted improvements in major outcomes related to the impact of fibromyalgia syndrome (FMS) on daily life, quality of life, depression symptoms, sleep regularity, sense of coherence, and pain [29], this secondary analysis focuses on selected items from the Short-Form Health Survey (SF-12) [31], Fibromyalgia Impact Questionnaire (FIQ) [32], and Bodily and Emotional Perception of Pain (BEEP) [33], which investigate symptoms attributable to the low energy/fatigue syndrome, a crucial aspect of FMs. Specifically, it examines Item 10 of the SF-12 regarding perceived energy; the ability to walk and the ability to climb stairs, Items 7 and 11 of FIQ; and the impact of pain on movement ability, Item 17 of the BEEP.

Owing to the crossover design employed, participants initially assigned to the control group (C) underwent observation as controls during the first parallel phase before transitioning to the experimental group where they received the intervention treatment. Consequently, the experimental group (E) was twice the size of the control group (C), with half of group E comprising participants from the preceding control group (C). Unlike in a complete crossover study, we chose not to transition participants from group E back to the control group (C) after the first phase. This decision was made due to the exploratory nature of the study as we were uncertain whether the observed effects would persist over time.

The study included assessments at two time points: T0 (0 weeks) and T1 (10 weeks). Additional general information regarding the trial is provided in the initial publication focusing on the main outcomes [29].

### 2.2. Sample

Sixty-four women with FMs were recruited from the Pain Unit of “Ospedale San Giovanni di Dio, Cagliari, Italy” (University Hospital of Cagliari). Inclusion criteria identified females over 18 years old who met the American College of Rheumatology criteria for the diagnosis of FMs [34], Exclusion criteria included the presence of intellectual disability and/or comorbid rheumatologic illnesses and being male. The almost entirely female prevalence of the disorder would have made male recruitment difficult and excessively prolonged the study timeline.

Participants were randomly assigned to two arms at a 1:1 ratio using computer-generated randomization. Codes were anonymized and masked.

The experimental group (E) underwent 10 sessions of heart rate variability biofeedback (HRV-BF) once a week in addition to receiving standard therapies as usual. The control group (C) received standard therapies as usual, including analgesics, antidepressants, and anticonvulsants, without a placebo. After the E treatment period, participants in group C transitioned to HRV-BF treatment in addition to standard therapies as usual and were subsequently included in group E.

### 2.3. Intervention Protocol

Blinding to group allocation was ensured for the evaluators. Participants were well informed that HRV-BF was not intended as a “cure” in the traditional sense but rather as a tool to aid in mitigating the short-circuit from awareness of pain vulnerability, which can induce increased tension and alertness, consequently exacerbating sensitivity to pain. The HRV-BF protocol was developed based on the existing literature [35], consisting of 10 sessions (1 session per week) lasting 50 min each. Sensors placed in the earlobe recorded HRV during these sessions, providing participants with real-time activity information. This visual feedback allowed participants to synchronize their breathing regularity with HRV patterns. Initially, a supervisor technician (a health-trained psychologist or “educator”) assisted during the first sessions to ensure the correct execution of breathing tasks. Over time, this support gradually decreased, empowering participants to become increasingly autonomous in self-inducing a state of relaxation. This acquired skill could then be extended to other “real” life contexts, particularly in facing stress or heightened pain. The mental health professionals involved in the project were qualified in the use of HRV technology and strictly adhered to the planned intervention protocol.

### 2.4. Evaluation Tools

The sociodemographic and main clinical characteristics of the sample were collected using a specific form, based on registered initial medical history and clinical reports. For this secondary analysis, we adopted Item 10 of the Short-Form Health Survey (SF-12) as the main outcome measure. This item assesses the respondent’s perception of energy levels over the past four weeks, with responses coded on a scale from 1 (“never”) to 6 (“always”). The SF-12, which has an internal consistency of Cronbach’s α = 0.94, is designed to evaluate an individual’s perception of quality of life [30]. In addition to Item 10 of the SF-12, Item 7 from the Fibromyalgia Impact Questionnaire (FIQ) [32], (“During the past week, have you been able to walk for a few blocks”) and Item 11 (“in the past week, how have you been able to climb stairs”) (FIQ) [32] were considered as a tool designed to assess the impact of fibromyalgia on daily life, with its internal consistency of Cronbach’s α = 0.90. The scale comprises 20 self-administered questions that explore three components: ability to perform daily activities, number of days of well-being in the last week, and work-related issues. Higher scores on the FIQ indicate a greater impact of fibromyalgia. Specifically, the scale asks participants to rate their ability to perform certain tasks over the past week, such as walking (Item 7) and climbing stairs (Item 11). Responses are coded from 0 (Always) to 3 (Never). We selected these two questions because they are relatively independent of the economic and cultural status of the women in our sample. For example, many women over 50 did not work due to adherence to a traditional Italian family model, while many young women were unemployed due to the economic crisis. Therefore, despite this tool being considered one of the gold standards for assessing the impact of the syndrome, questions related to work efficiency could introduce bias into the assessment. From the Bodily and Emotional Perception of Pain (BEEP) [33], Item 17 was considered (“How much has pain limited your ability to move”). The BEEP, a tool consisting of 23 items [33], assessed the perception of pain linked to the emotional impact and pain’s interference with daily life, mood, relationships, and social rhythms, with its internal consistency of Cronbach’s α = 0.92. The higher the scores, the higher the perception of pain and limitations.

### 2.5. Statistical Analysis

The distributions under evaluation were assessed using the Kolmogorov–Smirnov test of normality. The change within groups over time (T0 vs. T1) for the analyzed items was calculated as the difference in the mean score ± standard deviation by one-way ANOVA for repeated measures given that all variables of interest are normally distributed. Subsequently, differences in score change between groups were measured using ANOVA. A comparison of nominal variables between groups was conducted using the chi-square test. All statistical analyses were performed using SPSS software (v. 28.0.1.0., IBM, Armonk, NY, USA), with a *p*-value < 0.05 considered statistically significant.

## 3. Results

The recruitment period started in May 2020 and was extended by nine months due to the restrictions imposed for the containment of the COVID-19 pandemic. The primary completion data were completed in July 2022. The sociodemographic and clinical profiles of the study groups are presented in Table 1. There were no significant differences observed between the two groups in terms of sex, age, education, employment status, or duration of illness and perception of pain at the baseline. Of the total participants enrolled in each group, 23 (71.9%) completed the trial in the experimental group, compared with 20 (62.5%) in the control group [χ2 = 0.638; 1df; *p* = 0.424].

Table 2 illustrates that the improvement in Item 10 of SF-12 was 12% higher in the experimental group compared with the control group (0.39 ± 0.31 vs. 0.15 ± 0.28, F = 7.010, *p* = 0.010), and the improvement in Item 7 of FIQ was 26.5% higher in the experimental group than in the control group (0.53 ± 0.27 vs. 0 ± 0.33; F = 35.444, *p* < 0.0001). However, the improvement in Item 11 of the FIQ did not show any statistically significant difference between the two groups. Additionally, Item 17 of BEEP demonstrated a gain of 4 in improvement when comparing the experimental group with the control group (0.17 ± 0.17 vs. 0.05 ± 0.19, F = 0.4779, *p* = 0.003).

## 4. Discussion

Data from this study suggest that HRV-BF may have specific efficacy on syndromic components of energy/fatigue in FMs and on the perception of pain related to simple activities. The feeling of energy, measured through Item 10 of the SF12 questionnaire, showed an improvement of 18.6% in the experimental group and of 6% in the control group. Similarly, individuals who underwent the experimental treatment exhibited a 26.5% improvement in the belief that they would be able to walk for a few blocks (Item 7 of the FIQ), whereas the control group showed no improvement. 

The BEEP item (17) referred to as how much the perception of pain limits simple activities improved with a statistically significant difference between the experimental and control groups, although this difference is of a small entity. Such similar improvement is not evidenced in Item 11 of the FIQ. However, in this case, it is the perception of a complex activity and not defined as an entity in the questionnaire adopted. That is, we do not ask how much we feel capable of climbing a defined number of steps or, for example, a floor, but simply whether we feel strong enough to climb steps (undefined in quantity). This obviously may require, in the imagination of those who respond, a higher level of energy and indefinite quantity compared with that required to walk (Item 7 FIQ) or if the pain interferes with the ability to move (Item 17, Beep). The divergence in the improvements in the four items examined is, therefore, not inconsistent but is in line with a moderate improvement, which does not reach to affect the response of FIQ Item 11, which evidently would require a greater improvement. To the best of our knowledge, few studies have highlighted a specific improvement in the effects of treatment on fatigue symptoms in fibromyalgia apart from sporadic anecdotal reports [36], specifically with use of biofeedback [35,37,38,39]. 

The intervention was well accepted and no side effects were documented; in the primary analysis [29], the dropout rate in the E group was similar to the C group and can be considered acceptable as the trial involved participants with chronic pain. In that kind of clinical population, the dropout rate during an intervention is usually much higher [40,41]. 

The main findings of the study from which this secondary analysis was drawn indicated that “The HRV-BF intervention did not demonstrate efficacy, making it difficult to speculate on the actual effectiveness of this technique” [29]; however, it is important to underline that it was a small-sample feasibility study conducted with the specific objective of studying the size necessary to obtain a study with sufficient power in Phase III.

The present secondary analysis highlights a possible relevant target in the use of the biofeedback technique adopted and suggests specific areas of attention in future studies. Furthermore, considering the commonality of fatigue syndrome in other disorders, such as chronic fatigue syndrome and chronic bipolar depression, it would be useful if particular attention was paid to the fatigue component by studies dealing with the use of biofeedback in these syndromes.

These study results, despite being novel concerning individuals with FMs, contribute to previous research experiences evaluating the effectiveness of biofeedback on fatigue in samples of individuals suffering from chronic fatigue syndrome [42,43] and, also, in certain clinical populations with anxiety and depression [44]. Several reviews have also evaluated these techniques for fatigue, highlighting encouraging results [45,46].

It should be considered that a notable portion of FMs participants in this study reported experiencing frustration due to encounters with various doctors, each proposing a different treatment, purportedly excellent and conclusive. This resulted in significant disappointment when no tangible results were achieved.

Hence, it was clearly communicated to participants that HRV-BF was not a “medical treatment” or a curative drug for the disease but rather the acquisition of a psychological regulation mechanism aimed at mitigating the cascade of catastrophic sensations often experienced by individuals vulnerable to pain. Incorporating psychoeducational aspects, especially in the early stages of interventions that utilize technology with vulnerable populations to prevent the person–machine relationship from becoming excessively alienating, could be a central aspect in evaluating the feasibility and effectiveness of technology used [47].

Individuals with FMs tend to feel weak when confronted with potential stress or pain, which, in turn, increases tension and, paradoxically, the pain response.

Our findings regarding specific items indicate that the anticipated “positive” response to HRV-BF treatment may unexpectedly affect the fatigue components more than the pain components, which is not surprising as the latter are typically emphasized in tools used to measure improvement in fibromyalgia. The underlying COVID-19 pandemic context in which the study was conducted adds significant value to the findings. These factors likely had a greater impact on individuals who, like those affected by FMS, were particularly vulnerable, and some authors have also suggested that long COVID syndrome may be linked to fibromyalgia [48,49].

Evidence suggests a general increase in fatigue symptoms in the community and among specific “at-risk” populations due to repeated lockdowns and the resulting disruption of social rhythms [50,51,52,53]. Therefore, emphasizing a specific focus on components related to fatigue in this syndrome could represent a central and pivotal element.

## 5. Limitations

As already stated in the presentation of the main results, this study has the following limitations: the absence of a placebo in the control group and a small sample size.

The pandemic context and the fear of “vaccine-induced” fatigue may have increased the placebo effect accompanied by the treatment and not balanced by a placebo in the control group.

## 6. Conclusions

The present secondary analysis highlights a possible relevant target in the use of HRV-BF and suggests specific attention to the component of fatigue in future studies on the efficacy of treatments for FMs. Furthermore, considering the prevalence of fatigue syndrome in other disorders, such as chronic fatigue syndrome and chronic bipolar depression, it would be beneficial for studies investigating the use of biofeedback in these syndromes to focus specifically on the fatigue component. This approach could help more effectively guide interventions aimed at addressing elements that significantly impact the lives of this specific vulnerable population.

## Figures and Tables

**Table 1:** Characteristics of the sample at T0 experimental group (E) (N = 32) and control group (C) (N = 32).

	E	C	Statistics	*p*
Sex (Females)	100%	100%		
Age	54 ± 9.2	56.2 ± 10	Anova 1,62df F = 0.839	0.363
Education (Middle school or higher)	15 (46%)	15 (46%)	Chi-square 1df = 0	1
Unemployed	5 (15%)	9 (28%)	Chi-square 1df = 1.283	0.257
Years in illness	16.1 ± 13.5	14.1 ± 9.3	Anova 1,62df F = 0.476	0.493
Beep	89 17.1	84 23.9	Anova 1,41df F = 0.634	0.430

Legend: T0: pre-treatment time (baseline); M ± S: mean and standard deviation; df: degrees of freedom; F: Statistic of ANOVA; p: *p*-value. Beep: Bodily and Emotional Perception of Pain.

**Table 2:** Difference by time (T0 vs. T1) and groups (experimental vs. control) of items concerning energy levels and fatigue over SF-12, FiQ, and BEEP.

E (N = 23); C (N = 20)	T0	T1	Difference	Improvement
E SF12 Item 10—Energy levels	2.09 ± 0.88	2.48 ± 0.97	0.39 ± 0.31	18.6%
C SF12 Item 10—Energy levels	2.25 ± 1.13	2.40 ± 1.15	0.15 ± 0.28	6.6%
Anova 1,141df Experimental vs. control			F = 7.010 *p* = 0.010	Difference 12%
E FIQ Item 7—Walking	2.00 ± 1.10	1.47 ± 1.34	0.53 ± 0.27	26.5%
C FIQ Item 7—Walking	1.5 ± 1.16	1.5 ± 1.36	0 ± 0.33	0%
Anova 1,141df Experimental vs. control			F = 35.444 *p* < 0.0001	Difference 26.5%
E FIQ Item 11—Climbing stairs	0.95 ± 0.99	0.65 ± 0.86	0.30 ± 0.25	No differences
C FIQ Item 11—Climbing stairs	1.35 ± 1.10	1.00 ± 1.44	0.35 ± 0.22	
Anova 1,141df Experimental vs. control			F = 0.478 *p* = 0.493	
E Beep item 17—Pain interference with mobility	3.69 ± 0.80	3.52 ± 0.80	0.17 ± 0.17	Difference 5%
C Beep item 17—Pain interference with mobility	3.85 ± 1.15	3.80 ± 1.16	0.05 ± 0.19	Difference 1%
Anova 1,141df Experimental vs. control			F = 0.4779 *p* = 0.0035	Difference 4%

Legend: E: experimental group; C: control group; T0: pre-treatment time; T1: post-treatment time; M ± S: mean and standard deviation; df: degrees of freedom; M ± SD: Mean, standard deviation; df: degrees of freedom; F: Statistic of ANOVA; *p*: the *p*-value.

## Data Availability

All data generated or analyzed during this study are included in this published article.
